# A genetic dissection of breed composition and performance enhancement in the Alaskan sled dog

**DOI:** 10.1186/1471-2156-11-71

**Published:** 2010-07-22

**Authors:** Heather J Huson, Heidi G Parker, Jonathan Runstadler, Elaine A Ostrander

**Affiliations:** 1 Cancer Genetics Branch, National Human Genome Research Institute, National Institutes of Health, Bethesda, Maryland, 20892, USA; 2 Institute of Arctic Biology, University of Alaska Fairbanks, Fairbanks, Alaska, 99775, USA

## Abstract

**Background:**

The Alaskan sled dog offers a rare opportunity to investigate the development of a dog breed based solely on performance, rather than appearance, thus setting the breed apart from most others. Several established breeds, many of which are recognized by the American Kennel Club (AKC), have been introduced into the sled dog population to enhance racing performance. We have used molecular methods to ascertain the constitutive breeds used to develop successful sled dog lines, and in doing so, determined the breed origins of specific performance-related behaviors.

One hundred and ninety-nine Alaskan sled dogs were genotyped using 96 microsatellite markers that span the canine genome. These data were compared to that from 141 similarly genotyped purebred dog breeds. Sled dogs were evaluated for breed composition based on a variety of performance phenotypes including speed, endurance and work ethic, and the data stratified based on population structure.

**Results:**

We observe that the Alaskan sled dog has a unique molecular signature and that the genetic profile is sufficient for identifying dogs bred for sprint versus distance. When evaluating contributions of existing breeds we find that the Alaskan Malamute and Siberian Husky contributions are associated with enhanced endurance; Pointer and Saluki are associated with enhanced speed and the Anatolian Shepherd demonstrates a positive influence on work ethic.

**Conclusion:**

We have established a genetic breed profile for the Alaskan sled dog, identified profile variance between sprint and distance dogs, and established breeds associated with enhanced performance attributes. These data set the stage for mapping studies aimed at finding genes that are associated with athletic attributes integral to the high performing Alaskan sled dog.

## Background

"Alaskan sled dogs" are a recognized population of dogs of Northern breed ancestry. They were specifically developed as working dogs to haul cargo-laden sleds across the Arctic terrain [1,2]. They served as humans' primary means of transportation, protection, and companionship in northern snow-dominated climates for many years. Indeed, the late 1800's to early 1900's was termed the "Era of the Sled Dog" due to the breed's dominating presence in polar exploration and the boom of the Alaskan gold rush [3]. While the Alaskan sled dog experienced a decline in popularity as more modern modes of transportation became accessible in northern climates, they have recently undergone a rediscovery with the birth of sled dog racing, beginning in the late 1930's [2,3]. Concomitant with this rebirth has been a transition from working class dog to high performance athlete. While not recognized by the American Kennel Club as a distinct breed, consistency in behavior has lead to them being informally referred to as a "breed". The long-term goal of this study is to understand the genetic underpinnings associated with both the genetic heritage and the elite athletic performance of Alaskan sled dogs.

The Alaskan sled dog is comprised of several different lineages, optimized for different racing styles (long or short distance) [4-6], and we hypothesize that each will have a unique breed composition. Thus, we sought to identify breed composition profiles associated with expertise at specific tasks. The identification of these breeds would not only set the stage for genome wide association studies (GWAS) aimed at finding the underlying genes, but could theoretically explain why the introduction of certain breeds enhance performance traits in the Alaskan sled dog, while others have disappeared from the genetic make-up of today's sled dog.

**Figure 1:**
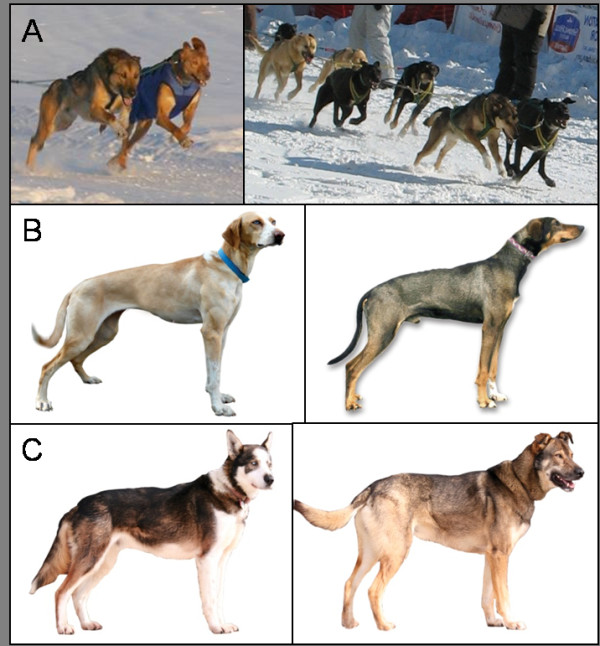
**Alaskan sled dogs are a mixed breed dog selected strictly for their racing performance**. A) Top row: Sprint racing teams of "traditional" Alaskan sled dogs (no purebred crossings in the last 5 generations) and Pointer crossed Alaskan sled dogs. Spandex dog coats (in blue) are commonly used on shorter-haired Pointer × Alaskan sled dogs when temperatures are ≤10'F. B) Middle row: Sprint sled dogs of 25% or greater Pointer ancestry according to their written pedigree records. C) Bottom row: "Traditional" Alaskan sled dogs from distance racing teams. All photos were taken between 2006-2009 of dogs competitively racing in high performance kennels.

The Alaskan sled dog is unique in that it is not confined to a breed standard of size or appearance, as are most AKC-recognized breeds. Rather, they are a mixed breed dog, with Northern breed ancestry, currently selected for high performance in sled dog racing. This selection for athletic ability has produced dogs of a particular physique. They are known for their quick, efficient gait, pulling strength, and endurance. Weight, averaging 55 lbs, and density of hair coat, vary depending upon racing style, geographic location, lineages, and cross breeding to purebred lines (Figure 1).

Sprint and distance sled dog racing are vastly different in terms of the distance traversed during a race and the speed at which this is accomplished. Long distance racing includes races of several hundred miles lasting multiple days, such as the Yukon Quest and Iditarod of over 1,000 miles in the subarctic winter[7,8]. Sprint racing is more analogous to track and field with multiple competition events defined by the size of the dog team [8,9]. The extreme differences in these racing styles, ranging from 30 miles in one day to 1,000 miles in less than ten days has lead to a divergence within the Alaskan sled dog population based on the essential physiological athletic attributes of endurance and/or speed as well as "work ethic," which encapsulates an animals' desire to perform. In this study we define the purebred dog profiles that have given rise to lineages of Alaskan sled dogs differing in their speed, endurance, and work ethic.

## Methods

### Sample Collection

One hundred and ninety-nine Alaskan sled dogs were sampled from eight "high performance" racing kennels. "High performance" sprint kennels are those whose dogs finished in the top 25% of sprint competitors in the International Sled Dog Racing Association [9] annual points standings. High performance distance kennels are those that had a primary team finish in the top 15% of all competitors for the Yukon Quest or Iditarod races during the two consecutive years that sample collection was undertaken [7,8]. Ninety percent of Alaskan sled dogs sampled from sprint racing kennels were from open and 8-dog racing classes with the remaining 10% competing in the 6-dog class. This was done to maintain consistency in the dogs sampled that they were being trained and raced at similar conditions of speed and distance based on their respective racing styles.

Prior to blood collection, all owners signed an informed consent document, consistent with NHGRI Animal Care and Use Committee rules. Whole blood samples were collected from the cephalic vein in 3-5 ml EDTA or ACD tubes. Dogs were sampled at their home kennels. Purebred dogs were sampled at AKC-sanctioned events. Samples were stored at 4°C prior to extraction, and genomic DNA was isolated using standard proteinase K/phenol extraction methods by Health Gene (Toronto, Canada) or RX Bioscience (Rockville, MD, USA). DNA samples were stripped of identifiers, numerically coded, and aliquoted for long-term storage at -70°C. Detailed pedigrees were collected for each individual sampled and entered into our database.

In addition to the Alaskan sled dogs, we sought to expand our reference data set of purebred dogs to be used for the comparison study. Towards that end, 44 purebred dogs, representing nine AKC breeds; Japanese Chin, Tibetan Spaniel, Anatolian Shepherd, Briard, Swedish Vallhund, Yorkshire Terrier, German Pinscher, Havanese, and English Springer Spaniel, were sampled and that data added to the existing data set of 132 breeds [10]. As we have done previously, only dogs who shared no common grandparents were selected for analyses [10,11]. Eight of the new breeds were represented by five unrelated individuals, while the German Pinscher breed was represented by three. Blood draw and sample preparation has been described previously [10,11].

### Performance Ratings

The sled dogs were rated on three aspects of performance: speed, endurance, and work ethic, using previously defined criteria specified for the distinct racing styles of sprint and distance [9]. The performance phenotypes and rating criteria were defined by one of us (H.H.) and reviewed by five professional and independent dog mushers. For both sprint and distance measures, five elite performers were selected for detailed study. In addition, we included five individuals from the other end of the spectrum who were consistently poor performers for each category The sprint racing category however had only three, two, and four representatives available for low speed, poor endurance, and poor work ethic, respectively. Distance racers had only four individuals sampled for low work ethic. Elite and poor performing dogs were compared to one another within racing style. Because of the extreme distance involved, distance dogs were each scored only one time during the calendar year. Sprinting dogs were scored on a weekly basis throughout training and racing season (approximately seven months) to assess consistency. However, the score collected in April, at the end of the season during peak performance, was used for this analysis. These performance scores accounted for the dog's overall performance throughout the entire year. Weekly scores for the sprint dogs were compared to the final score given to monitor consistency.

Speed was defined as an individual dog's ability to successfully maintain the necessary speed of the team. A dog was ranked 1 if it was capable of maintaining the speed of the team; 18-25 mph for sprint dogs and 8-12 mph for distance dogs; or ranked 2 if it was unable to maintain the required speed. Speeds were based on the performance levels of the kennels represented in the study.

Endurance was broken into three ranks; dogs were capable of covering the required mileage in good or poor condition (rank 1 or 2, respectively), or they were unable to finish the required mileage (rank 3). Mileage requirements ranged from 8-30 miles for sprint dogs and 991-1,150 miles for the distance sled dogs and were set according to race length requirements.

**Figure 2:**
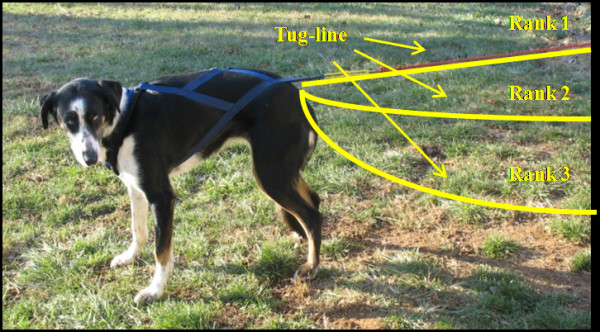
**Work ethic was scored on a three-tiered system based on the dog's willingness to run**. The effort a dog put forth during a run was determined by the amount of tension a dog placed on their individual tug-line. The tug-line, indicated with yellow, is the line attaching the dog's harness into the main line connecting the dog team to the sled. Dogs demonstrating the strongest effort, defined by having a constant tug-line tension throughout the run, were designated as rank 1 (top line). Rank 2 (middle line) defined dogs that had intermittent tug-line tension throughout the run, but maintained the speed of the team. The poorest performers, rank 3 (bottom line), showed no tug-line tension during the run but were capable of the speed and mileage. Dogs were not penalized due to the affects of injury.

The final trait, work ethic, was based on the dog's willingness to run, and was defined by the "effort" the dog displayed to pull the sled throughout the run. Effort was determined by the amount of tension a dog placed on their individual tug-line. The tug-line is the point at which the dog's harness attaches into the main line connecting the team to the sled. A three-tiered system was used in scoring (Figure 2). Dogs who scored a 1 showed the strongest effort, as evidenced by having constant tug-line tension throughout the entire run. Dogs scoring a 2 had occasional tension throughout the run, but maintained the speed of the team. The poorest performers, with a score of 3, showed no tension in the tug line throughout the run, but were capable of the speed and mileage. Dogs were not penalized in their rankings due to the affects of injury.

### Microsatellite Genotyping

A panel of 96 previously-described microsatellite-based markers were genotyped using DNA isolated from all dogs [11]. A clustering algorithm from the program STRUCTURE was used to both differentiate dog breeds as well as establish breed composition for the Alaskan sled dogs. Data from 132 previously genotyped breeds (5 dogs/breed) were included in this study [10,11] as well as the nine new breeds, described in the Methods, for a total of 141 purebred breeds.

One hundred-ninety nine Alaskan sled dogs were genotyped with the same 96 microsatellite-based markers. This included 116 dogs from four sprint racing kennels and 83 dogs from four distance racing kennels. Dogs were chosen for even distribution among all eight kennels, unrelated through the grandparent generation, in order to maximize the number of lineages tested. Dogs were also selected so that approximately equal numbers of high and low performers were included.

PCR amplification of microsatellite markers was done using a protocol similar to that published previously [11], but with the following slight modifications: 1 μl of 1.0 mM dNTP's, 0.1-0.2 μl of 10 μM forward and reverse primers, 0.315-0.42 μl of 50 mM MgCl, 0.05 μl TaqGold, 1 μl of 10× TaqGold buffer, 0.1 μl of 10 pmol/μl of M13 primer covalently linked with either 6FAM, VIC, NED, or PET fluorescent dyes (ABI), and 5 ng genomic DNA. Amplification was done at 95°C for 10 min, followed by 35 cycles at 94°C for 30 sec, 55°C or 58°C for 30 sec, and 72°C for 30 sec, followed by 10 min at 72°C. Samples were denatured in Hi-Di formamide with 15 pmol of GeneScan-500LIZ size standard (ABI, Foster City, CA). All samples were run in either 96 or 384 well plates with a positive and negative control on an ABI 3730×l capillary electrophoresis unit. Genotypes were called using GeneMapper 4.0 (ABI). All genotyping calls were checked manually with a positive control to assure consistent allele binning.

### Statistical Analysis of Population Structures

Population structure was assessed based on an allele frequency model using the program STRUCTURE at 100,000 iterations after a burn-in of 20,000 iterations [12-14]. K represents the number of populations assigned during each clustering run. Each run was repeated five times with populations being manually determined by breed membership, and then averaged over all runs. Population representatives of the purebred dogs and Alaskan sled dogs were unrelated through the grandparent generation. An optimum of five individual dogs were chosen to represent individual Alaskan sled dog lineages, however, in a few cases, only two dogs were available and both were thus used. These numbers are consistent with the number of individual dogs representing AKC breeds in the clustering analyses established by Parker et al [10,11]. The population clustering values of each individual representative sled dog were averaged for an overall breed composition of the specified sled dog population. Cluster analyses were run on 30 datasets of the Alaskan sled dogs and the purebred breeds to determine population representatives, breed composition profiles, and ancestral origins (Additional file 1: Table S1). These datasets varied by grouping dogs from sprint and distance racing kennels, the performance rankings of the individual dogs, and the number of domestic breeds represented. One particular dataset of 42 sprint dogs who were unrelated at the grandparent generation and 42 similarly unrelated distance dogs were used for the analysis of population structure for all Alaskan sled dogs. Alaskan sled dog population representatives were determined by choosing five population members from the 84 unrelated dogs with a population score of ≥ 0.9 within that population. Performance ability was not a factor when determining the individuals representing each dataset used for population structure.

### Breed Composition

Component breeds of the Alaskan sled dog were identified using the previously defined microsatellite-based markers [11]. One hundred forty-one purebred dog breeds were genotyped to ascertain the subset most closely related to the Alaskan sled dog. The analysis was restricted to two populations (K = 2), assigned based on similar allelic patterns. The data set also included 84 Alaskan sled dogs. The Alaskan sled dogs and purebred breeds of similar heritage clustered into one population. The second population consisted of purebred breeds with the most divergent allelic patterns to population one. The breeds that had a minimum of two out of five possible individuals clustered with the Alaskan sled dogs, and had a population score of ≥ 0.3, were utilized for the subsequent Alaskan sled dog breed composition analyses.

### Inbreeding Coefficients

Inbreeding values and heterozygosity were calculated with the Genetic Data Analysis (GDA) software using the microsatellite data [15]. Similar dataset groupings were used for the inbreeding analyses as had been used for the cluster analyses. All 141 breeds were investigated with and without the Alaskan sled dogs. The Alaskan sled dogs were analyzed as a single population of 84 unrelated dogs or as two independent sub-sets of 42 unrelated sprint dogs and 42 unrelated distance dogs, as previously described. Lastly, the sub-populations of sled dogs were compared. GDA established inbreeding coefficients termed f and theta-P, which are the equivalents of F_IS _and F_ST_, respectively and referred to as such hence forth. F_IS _represents the inbreeding of an individual relative to the subpopulation and F_ST _represents the inbreeding among subpopulations relative to the total population. Sigma-G represents the variance of alleles within individuals. Expected (H_E_) and observed (H_O_) heterozygosity and the mean number of alleles per locus (A) were also calculated.

## Results

### Alaskan Sled Dog Breed Identification

Previously, Parker et al. showed that with few exceptions, individual purebred dogs are correctly clustered by breed in an unsupervised clustering analysis using genotype data from just 96 microsatellite-based markers analyzed using the program STRUCTURE [10,11]. When the allowed number of clusters is restricted, reproducible groups of breeds are formed, typically encompassing breeds of similar appearance and shared heritage.

There was a 1% marker failure rate of the microsatellite-based genotyping calls for the combined 132 breeds previously genotyped by Parker et al. and the additional nine breeds in this study [10,11]. The Alaskan sled dogs had a slightly higher marker failure rate at 2.42% with less than a third of the total number of dogs in comparison to the domestic breeds. The highest marker failure rate within an individual Alaskan sled dog was 16%. Twenty-one percent of the domestic breeds had an individual marker failure rate greater than 16%. Only 4% of the domestic breeds had a failure rate higher than 40% (peaked at 61% marker failure seen in one individual).

We first compared the Alaskan sled dogs to 141 domestic dog breeds to determine the subset of breeds that had contributed most to the development of the sled dog. All dogs were genotyped using a set of 96 previously described microsatellite-based markers [11]. To determine the subset that were most related to the Alaskan sled dogs, we ran STRUCTURE using the parameter K = 2, to assign two populations for the cluster analysis. This placed all sled dogs into one population and most of the domestic breeds into a second. A small subset of domestic breeds showed significant clustering with the sled dogs, and were considered likely contributors to the population. These were used in subsequent breed composition analyses. Sixteen recognized breeds were identified with a population score of ≥ 0.3 within the sled dog population, which included both sprint and distance racing dogs. We next analyzed the sprint and distance dogs separately. Five additional domestic breeds were identified when comparing just the 42 unrelated sprint dogs to the 141 domestic breeds. However, analysis of the 42 unrelated distance dogs versus the 141 breeds did not reveal any additional related purebreds. In total, then, 21 domestic breeds were identified with a population score of ≥ 0.3 within the Alaskan sled dog population and are hence forth referred to as the "related breeds" in all future analysis.

The 21 "related breeds" included the Alaskan Malamute and Siberian Husky, which were expected based on historical information, and the Pointer, which has recently and repetitively been bred into the population [16]. The Samoyed, Chow Chow, and Akita also have historical roots as northern draft dogs [17]. Other breeds included in the "related breeds" group were the Saluki, Afghan Hound, and Borzoi, which are well known for their speed, the Great Pyrenees and the Anatolian Shepherd, both of whom are northern climate guard dogs, and the Weimaraner, a hunting breed of shared ancestral heritage to the Pointer [17]. Additional related breeds were the Japanese Chin, Shar-Pei, Shiba Inu, Shih Tzu, Pekingese, Lhasa Apso, Basenji, Tibetan Spaniel, and Tibetan Terrier, most of whom share an Asian heritage with the exception of the Basenji [10].

**Figure 3:**
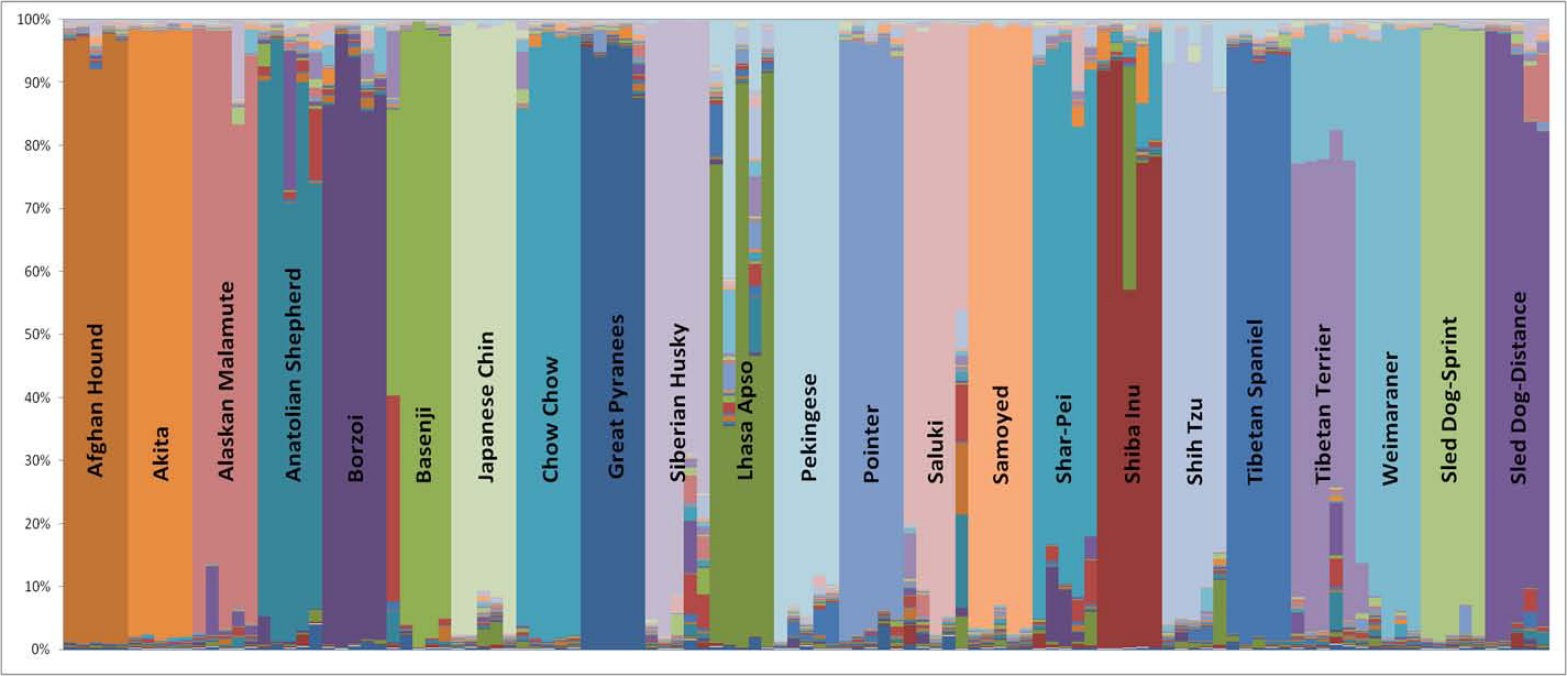
**Population structure of purebred dogs and Alaskan sled dogs**. The cluster analysis of 21 purebred breeds [10,11] and two Alaskan sled dog populations grouped by the racing style (sprint or distance). For each breed we utilized DNA samples from five individuals who were unrelated at the grandparent level. Individuals grouped into breed-specific clusters, denoted as differring colors on Figure 2, based on the percentage of their allelic pattern belonging to the specific cluster. The two Alaskan sled dog groups created their own populations based on their unique genetic signature of microsatellite-based markers.

In order to determine the breed composition of the Alaskan Sled dog, we compared their genotype data in a cluster analysis to that from the "related breeds" using STRUCTURE. Strikingly, when the number of populations allowed (K = 21) was equal to the number of domestic breeds in the analysis, sled dogs did not align with any specific breed, but rather defined their own breed group (Figure 3). Interestingly, the Alaskan Malamute and Siberian Husky often grouped as a single breed, as did the Chow Chow and Shar-Pei. These data suggest, therefore, that the breed signature of the Alaskan sled dog is more distinct then a subset of breeds of similar heritage. Individual sled dogs ranged from 40-90% in terms of their Alaskan sled dog signature, while the remainder of each profile was a mixture of the 21 other breeds. These results establish the Alaskan sled dog as a breed, distinguishable by its genetic profile, regardless of the population's diversity in appearance and it's mottled history.

**Figure 4:**
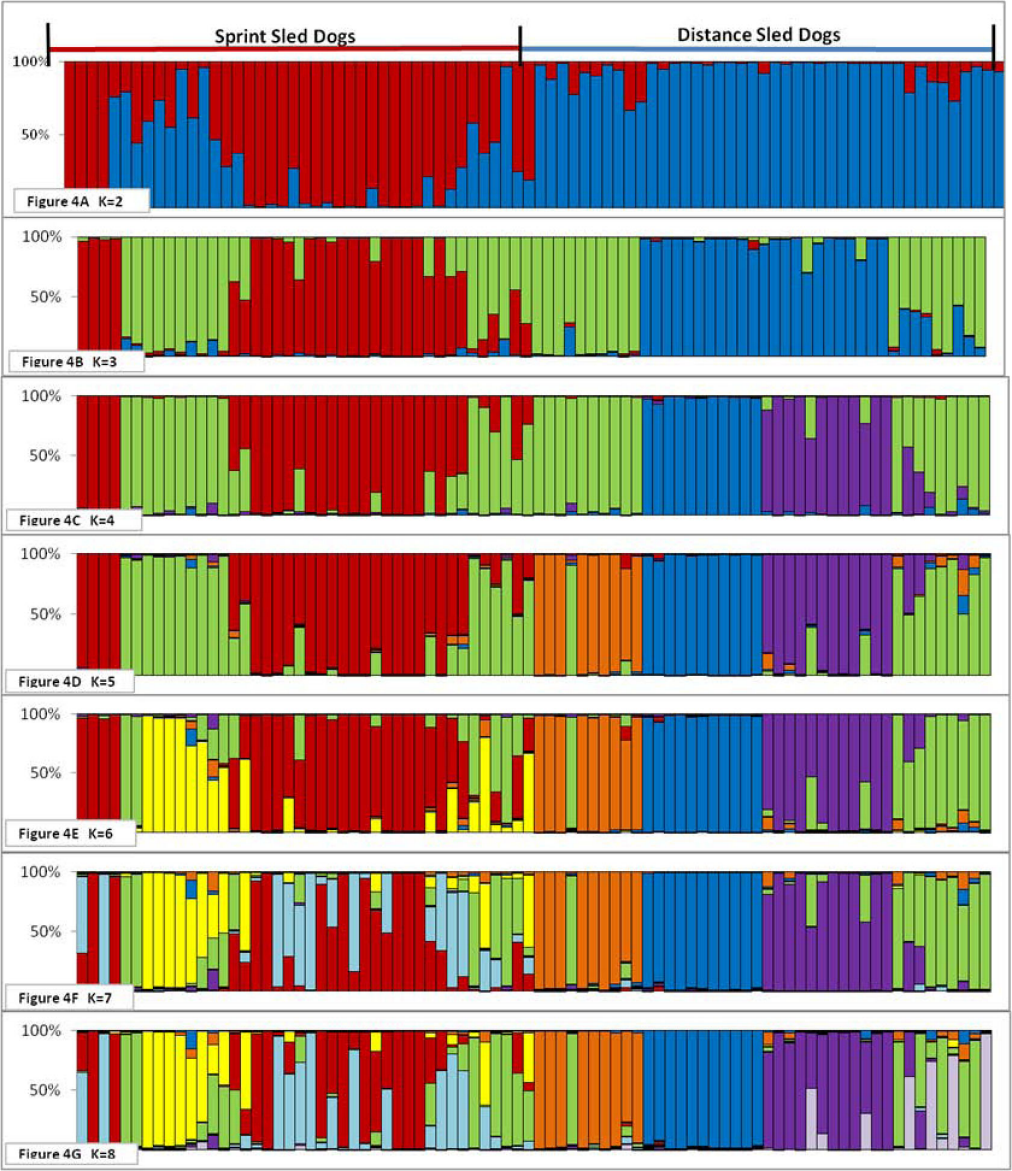
**Population structure of sprint and distance sled dogs during successive increase in assigned population numbers**. The population structure of 84 unrelated Alaskan sled dogs of even distribution between four sprint and four distance kennels. The 42 Alaskan sled dogs from the sprint kennels are on the left side of the figure and the 42 Alaskan sled dogs from the distance kennels are on the right side of the figure. Each population is designated by a different color in the chart. Individuals are categorized based on the percentage of their allelic pattern belonging to each of the populations. Figures 4A-G show a successive increase in the assigned number of populations from K = 2 through K = 8. In total, eight sub-populations, four in sprint dogs and four in distance dogs, were documented from the sampled Alaskan sled dogs.

Upon further analysis, the Alaskan sled dogs further separated into two clusters based solely on their racing style; sprint versus distance. This can be seen when representatives of both racing styles are analyzed with the domestic breeds (Figure 3) as well as when they are analyzed independently (Figure 4A). Five dogs, displaying the most distinctly uniform allelic profiles associated with each racing type (sprint versus distance), and referred to as the "extreme" representatives for each style, were then selected for further analysis.

### Ancestral Groupings

**Figure 5:**
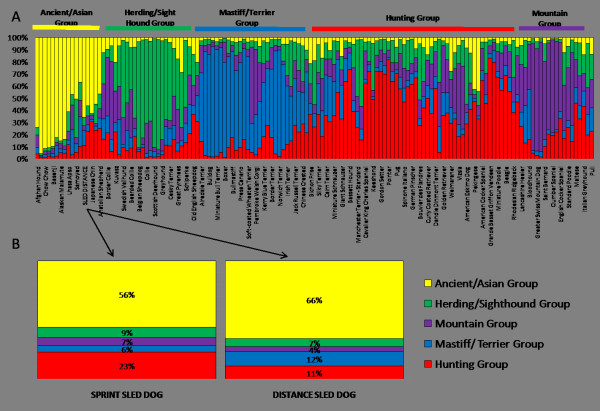
**Ancestral breed clustering and composition differences of the sprint and distance Alaskan sled dogs**. A). The five ancestral breed clusters signified by distinct colors as determined by Parker et al in 2004, 2007. One hundred forty-one purebred breeds (132 breeds from Parker et al with 9 new breeds) and sprint and distance populations of Alaskan sled dogs are represented in the clusters. Both Alaskan sled dog populations repeatedly cluster among the Ancient/Asian Group (yellow). B). The sprint and distance sled dog populations were investigated for ancestral group composition differences. We observe an increase in the Hunting Group (red) contribution among the sprint dogs and an increase in the Mastiff/Terrier Group (blue) contribution among distance dogs.

We investigated the specific relationship between sled dog populations and purebred dog breeds for clustering based on ancestral heritage. In addition, we looked at the composition of sprint and distance dogs with regard to the five major ancestral clusters defined by Parker et al [10,11]. An Ancient/Asian group, together with a Herding/Sight hound, the Mastiff/Terrier, Hunting and Mountain groups were previously determined as the most probable clusters from an analysis of 132 breeds [10,11]. In this more recent analysis of 141 breeds, both the sprint and distance populations consistently clustered within the Ancient/Asian group (Figure 5A). The Ancient/Asian group is the first of the populations to distinguish itself at K = 2 when all 141 purebred breeds are analyzed. The clustering of the Alaskan sled dogs with the Ancient/Asian group may be attributed to a number of factors. However, the fact that the Alaskan Malamute and Siberian Husky are both members of the Ancient/Asian group and are the primary purebred breed components of the Alaskan sled dogs may be influential. The breed membership to this cluster also illuminates why such unlikely breeds as the Lhasa Apso, Pekingese, and Shih Tzu which are also members of the Ancient/Asian group were found within the related breeds to the Alaskan sled dog.

We identified a generalized breed composition for the sprint and distance dogs based on their membership in these ancestral source groupings (Figure 5B). The higher percentage of breed composition attributed to Alaskan Malamute and Siberian Husky within distance sled dogs accounts for a higher clustering value of the distance sled dogs within the Ancient/Asian group. The sprint sled dogs owe a higher portion of their group composition to the Hunting group with a small increase in variation to the Herding/Sight hound group. In contrast, the distance sled dogs have a slight increase in the Mastiff/Terrier group.

### Population Structure and Breed Composition

We next examined the population structure within the two subgroups of Alaskan sled dogs. Clustering analysis of each racing style produced four sub-populations (Figure 4). The four distance sub-populations were kennel-specific. Three of the four fell out as distinct populations before any of the sprint sub-populations (K = 5) (Figure 4. This suggests that the distance-associated populations are genetically more distinct from one another then are any subset of sprint dogs. The last distance population to separate from the large cluster of sprint dogs was, interestingly, the most successful kennel sampled, as defined by number of wins (Figure 4G). The individual dog's performance was not based on winning percentage of the kennel.

**Figure 6:**
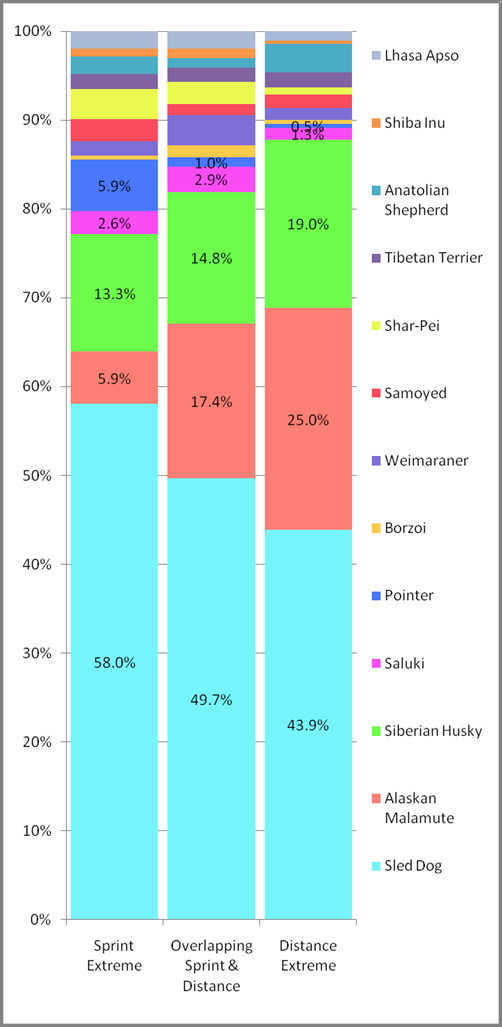
**Breed composition of Alaskan sled dogs reflected by three populations based on racing style**. The three populations represented extreme sprint, extreme distance, and a 3^rd ^overlapping population of sprint and distance sled dogs. The Alaskan sled dogs are assigned to three populations based on clustering analysis of microsatellite-based markers that are used to establish breed composition of each group. The percentage of each breed is denoted by a different color. The left most group is comprised of ten individuals representative of the "extreme" sprint sled dogs; the right most group is comprised of ten individuals representative of the "extreme" distance sled dogs; and the middle group is comprised of the remaining ten sprint and ten distance sled dogs which cluster together. There is an overall trend for increased Alaskan sled dog, Pointer, and Saluki signature in sprint sled dogs and an increase in Alaskan Malamute and Siberian Husky signature seen in distance sled dogs.

By comparison, the four sprint dog populations do not align well with kennel of origin. At K = 6 (Figure 4E) the sprint population divides into a major and minor group. At K = 7 and 8, two additional populations are defined. Interestingly, at K = 8 the distance population that was revealed last had some representation in the final sprint group. This suggests that the most successful distance dogs retain some genetic features of sprinters. We next determined which AKC recognized breeds accounted for the majority of the Alaskan sled dog signature (Figure 5). To do this, we evaluated the three groups as they clustered at K = 3 in Figure 4B; the extreme sprint dogs, extreme distance dogs, and the remaining overlapping sprint and distance sled dogs. At K = 22, we found that the sprint dogs had the largest signature for Alaskan Sled Dog (58%) (Figure 6, Column 1). They also had the largest signature for Pointer (5.9%), and the smallest signature for Alaskan Malamute (5.9%) and Siberian Husky (13.3%). By comparison, the extreme distance dogs (Figure 6, Column 3) had the weakest signature for Alaskan Sled dog (43.9%) and the largest signature for Alaskan Malamute (25%) and Siberian Husky (19%). They also had the smallest signature for Pointer (0.5%). As expected, the group that overlapped sprint and distance (Figure 6, Column 2) had a composite profile. However, they did have the largest component of Saluki (2.9%) in comparison to that observed in the extreme sprint (2.6%) and extreme distance (1.3%) dogs.

**Figure 7:**
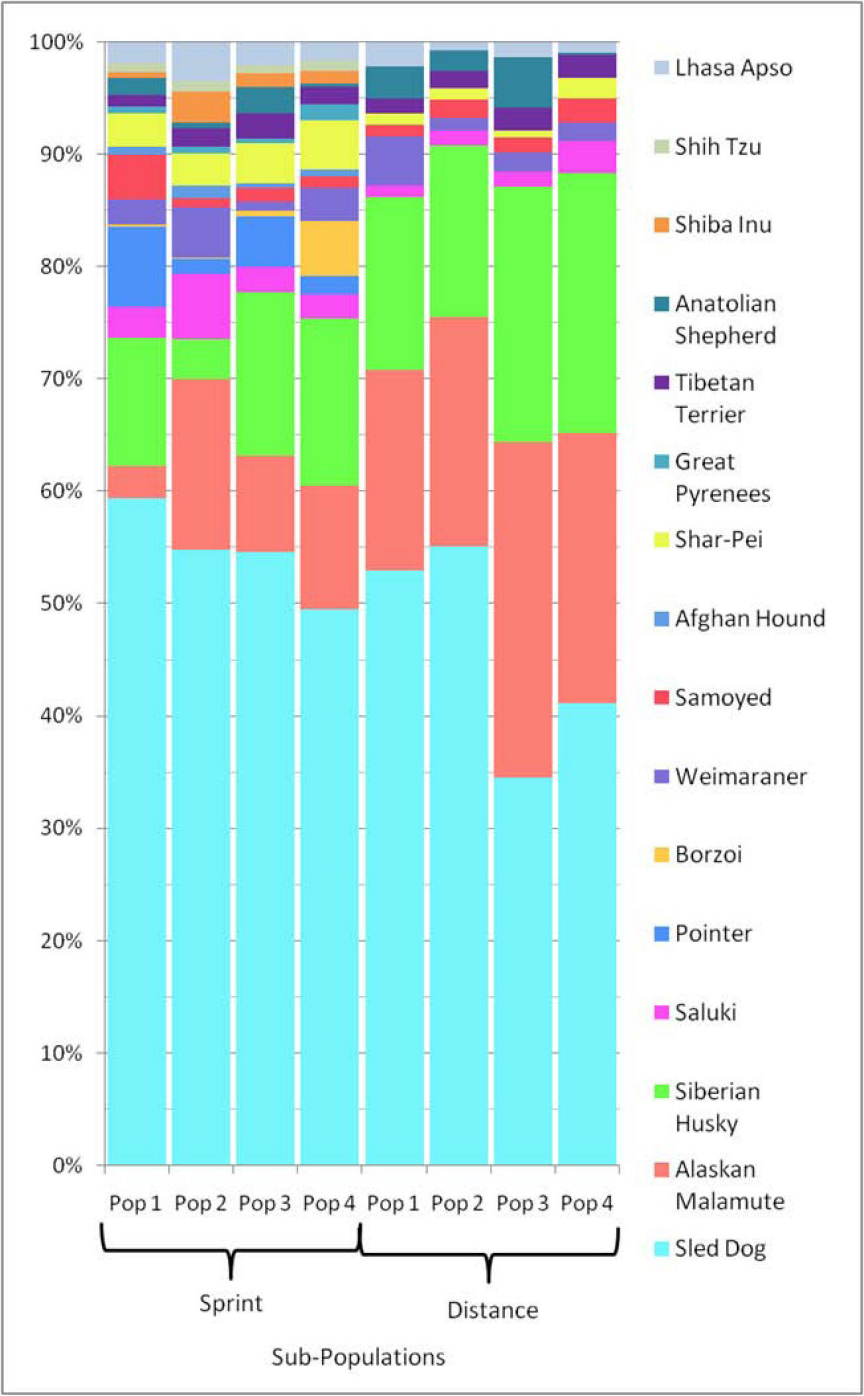
**Breed composition differences between Alaskan sled dog sub-populations**. Four sprint sub-populations and four distance sub-populations were identified through cluster analysis (Figure 4G). These sub-populations were analyzed for breed composition differences as depicted. Each component breed is represented by a unique color (which corresponds to colors used in Figure 6). The sprint sub-populations illustrate the greatest differences in the Saluki, Pointer, and Weimaraner.

We further refined our analysis, by analyzing the purebred breed composition of each of the eight populations defined in Figure 4. At K = 22, we observe that breed composition differences among the sub-populations highlighted specific trends (Figure 7 & Table 1). The four distance sub-populations showed the greatest variation in terms of Alaskan sled dog, Alaskan Malamute, and Siberian Husky contribution. Distance sub-population one did show a slightly greater contribution from the Weimaraner than did the other distance populations, and all showed some variation in terms of Anatolian Shepherd contribution. By comparison, the sprint sub-populations all showed smaller degrees of variation across a wider range of purebred breeds. Individual sprint sub-populations displayed a particular influence of specific breeds, such as the Pointer in sub-population 1 and 3, Saluki in sub-population 2, and Borzoi in sub-population 4. The refinement of breed composition down to the sub-population level allows for a more focused interrogation of sled dog attributes acquired from specific breeds.

**Table 1:** The percentage breed composition of four Alaskan sled dog sprint sub-populations and four Alaskan sled dog distance sub-populations.

	Sled Dog	Alaskan Malamute	Siberian Husky	Saluki	Pointer	Borzoi	Weimar-aner	Samoyed	Afghan Hound	Shar-Pei	Tibetan Terrier	Anatolian Shepherd
**Sprint Sub-Populations^A^**												
**Pop 1**	59%	3%	11%	3%	7%^B^	0%	2%	4%	1%	3%	1%	2%
**Pop 2**	55%	15%	4%	6%^C^	1%	0%	4%^D^	1%	1%	3%	2%	1%
**Pop 3**	55%	8%	15%	2%	4%^B^	1%	1%	1%	0%	4%	2%	2%
**Pop 4**	50%	11%	15%	2%	2%	5%^E^	3%	1%	1%	4%	2%	0%
**Distance Sub-Populations^A^**												
**Pop 1**	53%	18%	15%	1%	0%	0%	4%^D^	1%	0%	1%	1%	3%^F^
**Pop 2**	55%	20%	15%	1%	0%	0%	1%	2%	0%	1%	1%	2%
**Pop 3**	35%	30%	23%	1%	0%	0%	2%	1%	0%	1%	2%	4%^F^
**Pop 4**	41%	24%	23%	3%	0%	0%	2%	2%	0%	2%	2%	0%

A. The breed composition of each sub-population is the average breed composition of the five most representative members of the sub-population based on haplotype pattern.

B. Sprint sub-populations 1 and 3 have the highest percentage of Pointer breed composition.

C. Sprint sub-population 2 has the highest percentage of Saluki breed composition.

D. Sprint sub-population 2 and Distance sub-population 1 have the highest Weimaraner breed composition.

E. Sprint sub-population 4 has the highest percentage of Borzoi breed composition.

F. Distance sub-populations 1 and 4 have the highest percentage of Anatolian Shepherd breed composition.

### Inbreeding

We next examined the degree of inbreeding in the sled dog populations compared to purebred dogs. Inbreeding and heterozygosity statistics were calculated using the same 96 microsatellite-based markers described previously and the software program GDA [15]. We observed, first, that inbreeding coefficients of F_IS _and F_ST _both demonstrate at least a five-fold higher inbreeding value for purebred breeds than the Alaskan sled dog sprint and distance populations combined (Table 2). This is further exemplified by the combined sprint and distance populations of Alaskan sled dogs having a 15% increase in allele variance (sigma-G) and twice the number of alleles per locus (A) than the average purebred breed. Observed heterozygosity (Ho) calculations of the sprint and distance populations analyzed together or separately, had a 15% increase compared to the average purebred breed. These data support the idea that Alaskan sled dogs are generally less inbred than purebred breeds (Table 2).

**Table 2:** Sled dogs were 5× lower in inbreeding and 15% higher in observed heterozygosity than purebreds.

Groupings	F_IS _^A^	F_ST _^B^	Sigma-G^C^	A^D^	He^E^	Ho^F^
**Purebred Breeds Only^G^**	0.1085	0.2538	41.8949	2.6064	0.4729	0.4299
**Purebred/Sprint/Distance^H^**	0.1055	0.2534	42.0894	2.6100	0.4738	0.4318
**All Sprint & Distance^I^**	0.0170	0.0514	57.5737	5.6927	0.6090	0.5962
**All Sprint**	-0.0034					
**All Distance**	0.0456					
**Unrelated Sprint & Distance^J^**	0.0292	0.0424	57.5158	5.3646	0.6162	0.5985
**Unrelated Sprint**	0.0061					
**Unrelated Distance**	0.0526					
**8 Alaskan Sub-Populations^K^**	-0.0554	0.1349	56.2292	3.0156	0.5581	0.5849
**4 Sprint Sub-Populations^L^**	-0.0729	0.1082	58.7893	3.1016	0.5749	0.6111
**4 Distance Sub-Populations^M^**	-0.0361	0.1422	53.7001	2.9297	0.5413	0.5586

A. F_IS _is the degree of inbreeding within populations (correlation of alleles within individuals within one sub-population).

B. F_ST _is the overall inbreeding coefficient (correlation of alleles of different individuals in the same population).

C. Sigma-G is the variance of alleles within individuals.

D. A is the mean number of alleles per locus.

E. He is the expected heterozygosity of a population.

F. Ho is the observed heterozygosity of a population.

G. Data set included 141 purebred breed populations (681 individual dogs).

H. Data set included 141 purebred breed populations and 2 Alaskan sled dog populations using the 5 most representative individuals of the sprint racing and distance racing styles (10 total Alaskan sled dogs).

I. Data set included all 199 Alaskan sled dogs with microsatellite data. Relatedness not accounted for.

J. Data set included 42 unrelated to the grand-parent generation Alaskan sled dog representatives of each racing style (sprint versus distance) (84 total Alaskan sled dogs).

K. Data set included the 5 most representative individuals of each sub-population found within the sprint and distance racing style (40 Alaskan sled dogs).

L. Data set included the 5 most representative individuals of each of the 4 sprint sub-populations (20 sprint Alaskan sled dogs).

M. Data set included the 5 most representative individuals of each of the 4 distance sub-populations (20 distance Alaskan sled dogs).

**Figure 8:**
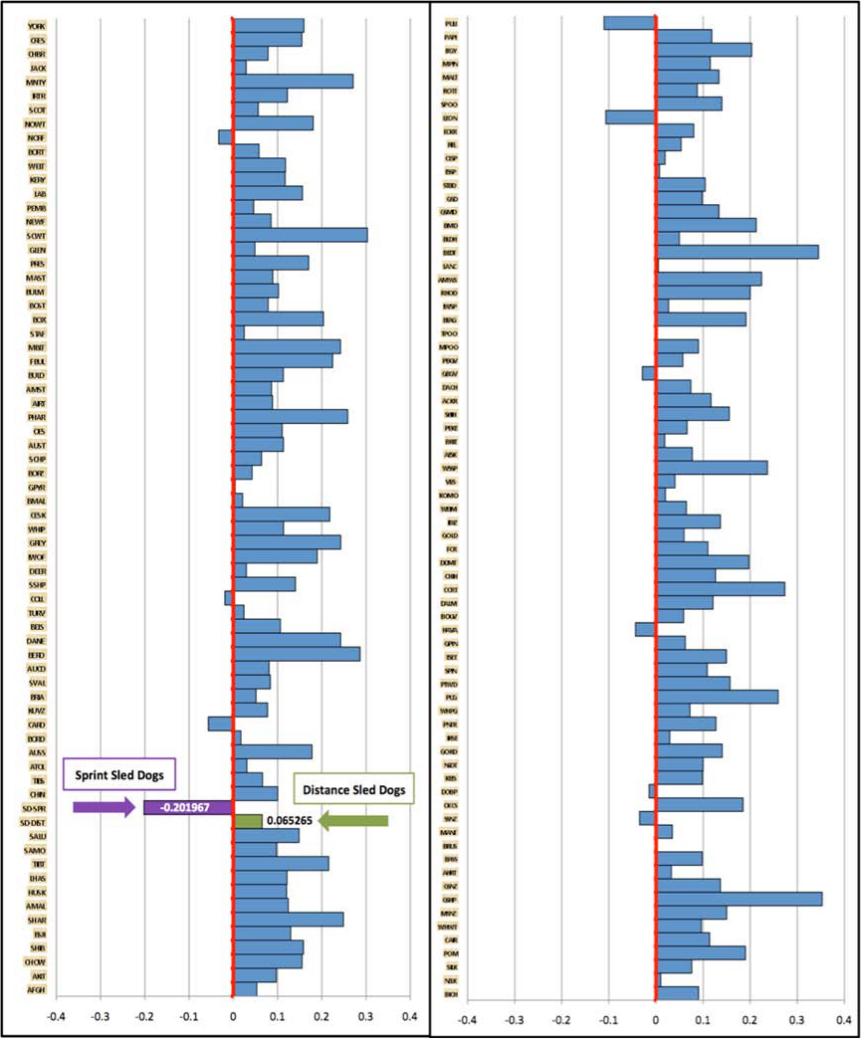
**The degree of inbreeding F_IS _within breed populations**. The degree of inbreeding, F_IS_, as determined for 141 purebred breeds (681 individuals, ~5 individuals per breed) and two Alaskan sled dog sprint and distance racing populations (five sprint and five distance individuals). The 141 breeds are listed in alphabetical order from top to bottom and are viewed in two identically scaled panels side by side. The vertical red line indicates the midpoint in Hardy Weinberg equilibrium, with inbreeding values ranging from -1 (below the red line) to +1 (above the red line). The more negative values indicate an excess of heterozygosity while the more positive values represent an excess of homozygosity. Each blue bar represents the degree of inbreeding for a single breed. The purple bar signifies the sprint dogs, and the green bar indicates the distance dogs. We observe an excess of heterozygosity within the sprint sled dogs.

When we compared the level of inbreeding amongst sprint and distance populations we observed that the inbreeding coefficient F_IS _was 8-fold higher for the distance population than the sprint population (Table 2). In Figure 8, we compare the individual F_IS _values for 141 purebred breeds with the individual values for sprint and distance populations. An excess of heterozygosity within the population is represented by a more negative value of F_IS_, whereas an excess of homozygosity is represented as a more positive value. Zero represents Hardy-Weinberg equilibrium. We observed that the sprint dog population (-0.20197 F_IS_) demonstrated an extreme excess of heterozygosity in comparison to the distance population (0.65265 F_IS_) and purebred breeds.

### Breed Contribution to Performance

In the final analysis we sought to determine how the introduction of various purebred breeds could enhance performance attributes. To accomplish this, each sled dog was rated on their individual skills with regard to speed, endurance, and work ethic using a set of defined criteria specified for the different racing styles (See Methods). Approximately five elite and poor performance representatives for each athletic attribute were analyzed in a cluster analysis with the "related breeds" to identify breed components associated with extremes of performance. The breed composition of elite and poor performance sled dogs was then compared to identify specific breeds that may enhance a performance phenotype (Table 3).

**Table 3:** The percentage change in Alaskan sled dog breed composition between high and low performing individuals.

Performance Phenotype	Racing Style	Sled Dog	Alaskan Malamute	Siberian Husky	Saluki	Pointer	Weimaraner	Samoyed	Anatolian Shepherd
**Speed**	**Sprint^A^**	5%^B^	-6%	-3%	3%^C^	-3%	1%	0%	0%
**Speed**	**Distance^A^**	25%^B^	-15%	-10%	-6%	-2%	1%	0%	3%^C^
**Endurance**	**Sprint^A^**	26%^B^	-10%	-7%	0%	-9%	-2%	0%	0%
**Endurance**	**Distance^A^**	-15%	11%^D^	11%^D^	2%	0%	0%	2%	0%
**Work Ethic**	**Sprint^A^**	38%^B^	-23%	-17%	-6%	-6%	-6%	2%	0%
**Work Ethic**	**Distance^A^**	11%^B^	-13%	-13%	0%	0%	0%	0%	6%^E^

A. The average breed composition of the five most representative dogs within the given race style for each athletic attribute.

B. There is an overall trend of increased Alaskan sled dog signature in higher performing dogs of all athletic phenotypes.

C. Saluki and Anatolian Shepherd show slight elevation for the speed phenotype.

D. Alaskan Malamute and Siberian Husky show an increase in representation within distance sled dogs for high endurance performance.

E. The Anatolian Shepherd is increased for the enhancement of the behavioral trait of work ethic in distance sled dogs.

The most striking observation was that strong performers in all categories and of both racing types showed a comparative increase in the Alaskan sled dog genetic signature. This was particularly illustrated by a 25% increase in the Alaskan sled dog signature seen in high performers of speed for distance dogs and a 26% and 38% increase in endurance and work ethic respectively for sprint dogs. In addition, endurance, which is obviously important in distance dogs, showed the highest increase of any AKC breed signature with an 11% increase in both Alaskan Malamute and Siberian Husky. This implies a significant role for sustaining the genetic integrity of the Alaskan Malamute and Siberian Husky in the distance sled dogs superior endurance performance.

A small number of breeds contributed disproportionately to elite performance of both racing types. Specifically, Saluki and Anatolian Shepherd had a 3% increase in their breed contribution for dogs exhibiting better speed in sprint and distance racing styles, respectively, compared to the 0% or negative affect observed otherwise. Also, the Pointer, which has been bred into sprint sled dogs in recent years with the idea of increasing speed, did not correlate with any athletic attributes. Finally, Anatolian Shepherd displayed a 6% increase for distance dogs with a high level of work ethic.

## Discussion

The Alaskan sled dog presents a case in which a genetically distinct breed of dog has been developed through the selection and breeding of individuals based solely on their athletic prowess. The creation of the Alaskan sled dog breed happened without the implementation of breed standards of size and appearance, or the closing of the breeding population to only those individuals deemed representative of the breed, as is the norm for AKC-recognized purebred breeds. We observed that inbreeding values were five-fold lower in the Alaskan sled dog population than the average purebred domestic breed. In addition, they demonstrated an excess of heterozygosity. These observations likely reflect the continual out-crossing for athletic enhancement that is common among Alaskan sled dogs. Interestingly, the process still led to the Alaskan sled dog repeatedly producing its own unique genetic signature. Indeed, the Alaskan sled dog breed proved to be more genetically distinct than breeds of similar heritage such as the Alaskan Malamute and Siberian Husky. Thus, we conclude that the breeding practices used to produce dogs of optimal performance have created a distinct breed of dog that developed using different criteria then are commonly used in the development and propagation of pure bred dogs.

Cluster analysis demonstrated that when the Alaskan sled dog population was compared to a large data set of purebred breeds they separated into two groups that align with their racing style, sprint versus distance. The same results were evident when the sled dog population was analyzed independently, without the purebred breeds. These two racing styles have diverged over the past 100 years [3] with athletic emphasis on either speed or endurance, as appropriate to the extreme differences in race length (sprint-30 miles, distance-1,000 miles). Unsupervised clustering analysis of the Alaskan sled dog population showed that 21% of a subset of 42 unrelated dogs from competitive sprint kennels grouped within the distance sled dog population. However, not a single individual from a distance kennel grouped within the sprint sled dog population. We speculate that a fraction of the dogs competitive in sprint racing are genetically capable of performing as distance dogs, but the reverse is not true.

In a clustering analysis with 141 purebred domestic breeds, both the sprint and distance populations consistently clustered within what has been termed the Ancient/Asian group of the five major ancestral clusters [10]. This supports the theory that at least a subset of early domesticated dogs originated in Asia and migrated with human nomadic tribes across the Bering Strait into North America [11,18-21]. Identification of compositional differences between the sprint and distance dogs with regard to the five ancestral clusters showed major differences. For instance, the Ancient/Asian group makes up 56% of the sprint and 66% of the distance group respectively. By comparison, the Hunting group comprises 23% of the sprint and 11% of the distance group. Finally the Mastiff/Terrier group also shows a two-fold difference with 6% associated with sprint while 12% comprise the distance population. We hypothesize that breeds from the Hunting group may enhance speed in sprint dogs and that breeds from the Mastiff group may contribute to the body stature and musculature necessary for the successful distance dogs.

Eight sub-populations, four sprint and four distance, were discovered within the Alaskan sled dogs. The distance populations were more genetically distinct, kennel specific, and had a higher average inbreeding value than the sprint populations. Interestingly, the final distance population to separate itself from the sprint populations is the most successful kennel in terms of race wins during the study period. The average F_IS _score of the sprint populations demonstrated the highest degree of heterozygosity when compared with the distance populations and 141 purebred breeds. We speculate that breeding programs are more confined within distance kennels while sprint kennel breeding programs cross between kennels. The inbreeding values support a higher level of gene flow among the sprint populations as well as a higher level of gene flow within the entire Alaskan sled dog population compared to the average domestic breed.

The dominant genetic signature found in all Alaskan sled dogs was that of the Alaskan sled dog breed. The extreme sprint population had a 14% higher average Alaskan sled dog signature and 5.5% higher Pointer breed signature than those of the extreme distance population. This suggests that the Pointer and the Alaskan sled dog signature itself play a major role in the enhancement of speed, which is the primary athletic attribute of sprint dogs. The Alaskan Malamute and Siberian Husky breed signatures were 19% and 6% higher respectively in the extreme distance dogs compared to the sprint dogs. This in turn, suggests that the Alaskan Malamute and Siberian Husky enhance endurance. Given their heavier build and stamina for cold temperatures this is not surprising.

A more detailed investigation of breed composition among the eight Alaskan sled dog populations revealed the particular influence of specific breeds. The four distance subpopulations differed primarily in terms of the contribution of the Alaskan sled dog, Alaskan Malamute, and Siberian Husky. In addition, Weimaraner and Anatolian Shepherd contributed to a few select sub-populations. Sprint sub-populations followed a different pattern with their primary breed component being Alaskan sled dog and their remaining composition consisting of smaller degrees of contributions from a wider range of purebred breeds. Individual sprint populations displayed a strong contribution from the Pointer, Saluki, Borzoi, and Weimaraner breeds.

The most compelling discoveries in the investigation of how various breeds may enhance performance attributes were seen with a 25%, 26%, and 38% increase in Alaskan sled dog breed for speed in distance dogs, and endurance and work ethic in sprint dogs, respectively. The highest contribution of a purebred was an 11% increase in both the Alaskan Malamute and Siberian Husky breed signatures for elite endurance performance in distance dogs as opposed to distance dogs who are known to exhibit poorer endurance abilities. This implies that strong genetic selection for Alaskan Malamute and Siberian Husky contributions has contributed to the elite performing distance dogs in terms of their endurance. The Saluki and Anatolian Shepherd both had minor positive influences for exceptional speed performance in sprint and distance populations, respectively. Unexpectedly, the Anatolian Shepherd, whose heritage describes a large, powerful, and independent northern livestock guardian dog, demonstrated a 6% positive influence in distance sled dogs of high work ethic. The Anatolian Shepherd's breed description of loyalty, independence, and hardiness portray a breed of similar character and strength to that of the distance sled dogs [17]. Interestingly, the Pointer, which has been repeatedly bred into the sprint dogs in recent years with the idea of enhancing speed was not found to positively affect speed performance. One hypothesis, is that the integration of Pointers with the sled dog population has not successfully enhanced speed performance. However, a comparison of four high profile sprint races' finish times in 1998 versus 2007, all showed faster completion times in 2007 [22]. There was a much lower degree of Pointer ancestry in the sled dog population in 1998, as compared to pedigree analysis of dogs in 2007 [23]. Another hypothesis that could explain the lack of significant Pointer contribution to speed may reflect a lack of significant difference in speed measures among the most and least successful sprint dogs. It may be that the contribution of the Pointer for speed is only evident when comparing sprint versus distance dog, or if more extreme representatives of elite versus less successful sprint dogs were assayed.

## Conclusions

We have identified component breeds of Alaskan sled dogs and defined specific breeds that have influenced the athletics of the dogs in terms of speed, endurance, and work ethic. It should be noted, however, that the crossing of purebred dogs into the Alaskan sled dog population does not guarantee performance enhancement in one generation. Rather, several generations of repeated selection and breeding of elite performers may be needed to permit optimal athletic performance to be obtained, as alleles of multiple genes are likely to play a role.

These experiments set the stage for genomic exploration into the phenotypes associated with successful sprint and distance performing Alaskan sled dogs. Whole genome association studies, currently underway, are likely to reveal genetic loci introduced by the various breeds that enhance each attribute of athletic performance. Such studies, are optimal for situations where there are a small numbers genes of major effect. We know little about the starting stock of Alaskan sled dogs and the number of major and or minor genes that contribute to these phenotypes. Current genomic tools, however, permit us to ask such questions, and our long term studies aim to paint a complete picture of the genetics of canine athletic performance, providing a complete palate of genes and alleles that contribute to these complex phenotypes.

## Authors' contributions

HJH generated the hypothesis and led the study design phase together with JR and EAO. Sample collection was conducted by HJH and JR. EAO and JR served as primary graduate mentors for HJH. Genotyping was completed by HJH for wet-lab portions. Statistical analysis was directed by HGP in collaboration with HJH. All authors were involved in scientific discussion of the project. HJH and EAO drafted the manuscript with all authors providing comments and final approval.

## Authors' information

HJH has 25 years of professional sled dog sprint racing and breeding experience.

## Supplementary Material

Additional file 1 **Table S1. STRUCTURE run input information including the population groupings, the number of populations designated, and the result objective**. Thirty datasets were investigated using the software program STRUCTURE in unsupervised cluster analyses. The datasets were categorized based on the purebred breeds and Alaskan sled dog populations utilized for exploring the ancestral origins, breed composition, and population structure of the Alaskan sled dogs. Click here for file
